# Experimental and translational models of Alzheimer’s disease: From neurodegeneration to novel therapeutic insights

**DOI:** 10.1016/j.tjpad.2026.100498

**Published:** 2026-01-30

**Authors:** Nadeemullah Khan, Somnath De, Suhasini Boddu, Navya Pravala

**Affiliations:** Department of Pharmacology, St. Pauls College of Pharmacy-Autonomous, Hyderabad, Telangana 501510, India

**Keywords:** *Alzheimer’s disease*, Amyloid-β (Aβ), Tau proteins, Neurofibrillary tangles, Amyloid precursor protein, Neuronal loss, Cognitive decline, Neurodegeneration, Translational models, Single-cell multi-omics, Neuroimaging

## Abstract

*Neurodegeneration on demand* represents a groundbreaking approach to modeling *Alzheimer’s disease* (AD) in animals, enabling precise study of its molecular and behavioral hallmarks. Novel techniques, including optogenetic activation of amyloidogenic pathways, viral vector-mediated delivery of mutated human genes (e.g., APP, MAPT), and synthetic tau fibril analogs, induce AD-like pathology, including amyloid-beta plaques, tau hyperphosphorylation, neuroinflammation, and synaptic loss in diverse species, ranging from transgenic rodents to cephalopods and cannies. Emerging platforms, such as bioengineered neural organoids grafted into immunocompromised hosts, allowed for the controlled onset of AD-like features, providing unique insights into disease progression. Advanced tools like real-time neuroimaging and single-cell multi-omics help elucidate the temporal and cellular dynamics of neurodegeneration. These models provided unparalleled opportunities to dissect AD’s complex mechanisms, including protein misfolding, glial dysregulation, and cognitive decline. However, challenges remained, including interspecies molecular disparities, incomplete replication of human AD complexity, and ethical concerns surrounding cognitive impairment in sentient models. This review explores these innovative strategies, their contributions to understanding AD’s pathogenesis, and their potential to accelerate the development of transformative therapies, while also addressing limitations and future directions for refining these pioneering models.

## 1 Introduction

*Alzheimer’s disease* is a progressive disease in which cells in the central nervous system malfunction or die leading to neurodegeneration. The condition is marked by the accumulation of Plaques composed of amyloid-beta and neurofibrillary tangles (NFTs) within the cerebral cortex, hippocampus, and limbic regions, following the classical Braak and Braak staging pattern, are recognized as the primary pathological features of the disease. Symptoms include memory damage or impairment, difficulties in decision-making, and diminished cognitive abilities. Projections indicate that the number of individuals suffering from Alzheimer’s-related dementia could rise to 152 million worldwide by 2050, with the most significant increases expected in lower middle-income countries [1]. In the United States, statistics from 2020 suggest that the number of Alzheimer’s patients aged 65 and older may surge from 5.8 to 13.8 million by 2050. Additionally, recent population studies in countries like Japan have revealed a significant rise in the prevalence of *Alzheimer’s (*AD) in recent years [2].

Various risk factors contribute to the progression and symptoms of *Alzheimer’s* (AD), as illustrated in Fig. 1. Consequently, there is a pressing need to develop effective treatment plans for individuals experiencing memory loss. Notably, the generation of amyloid-β (Aβ) from the *APP* i. e, precursor protein of amyloid and the formation of *NFT*s from tau protein (hyperphosphorylated) are the primary pathological features of AD were showed in Fig. 1A. As a result, Aβ and tau serve as key biomarkers for the disease. However, it is important to note that healthy individuals may exhibit these biomarkers at normal or elevated levels without exhibiting any signs or symptoms of AD, which complicates the challenge of making a presymptomatic diagnosis [3], [4].

**Fig. 1:**
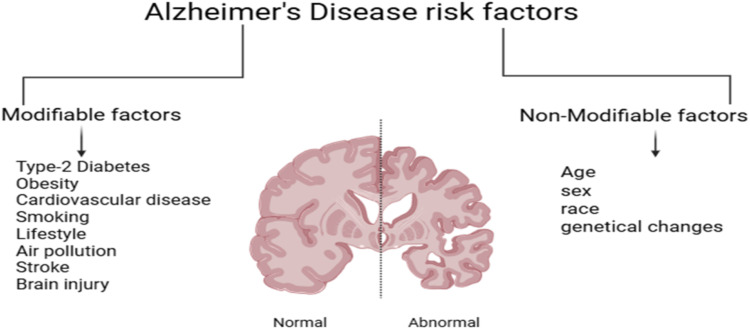
Potential risk or threat factors for *Alzheimer’s disease,* Schematic representation of major genetic, metabolic, and environmental factors contributing to AD.

**Fig. 1A:**
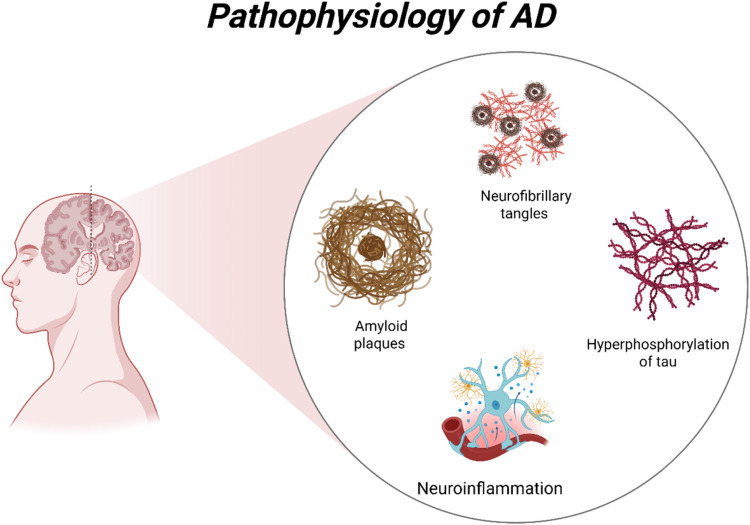
Comprehensive Pathophysiology of Alzheimer’s Disease**,** showing amyloid cascade, tau hyper phosphorylation, neuroinflammation**.**

The development of amyloid plaques, primarily composed of Aβ peptide, is a major pathological hallmark of Alzheimer’s disease (AD). According to the amyloid cascade hypothesis, Aβ accumulates and aggregates in the brain during the early stages of dementia, thereby initiating neurodegeneration. *Aβ* peptides are 37–43 amino acids in length, generated by proteolytic cleavage of amyloid precursor protein (APP) through sequential actions of β- and γ-secretases. In the **non-amyloidogenic pathway**, amyloid precursor protein (APP) is cleaved by **α-secretase** (primarily ADAM10 or ADAM17) within the Aβ domain, releasing **soluble APP-α (sAPPα)** and a membrane-bound fragment known as **CTF-α (C83)**. This cleavage precludes the formation of full-length amyloid-β peptides.

**Fig. 2:**
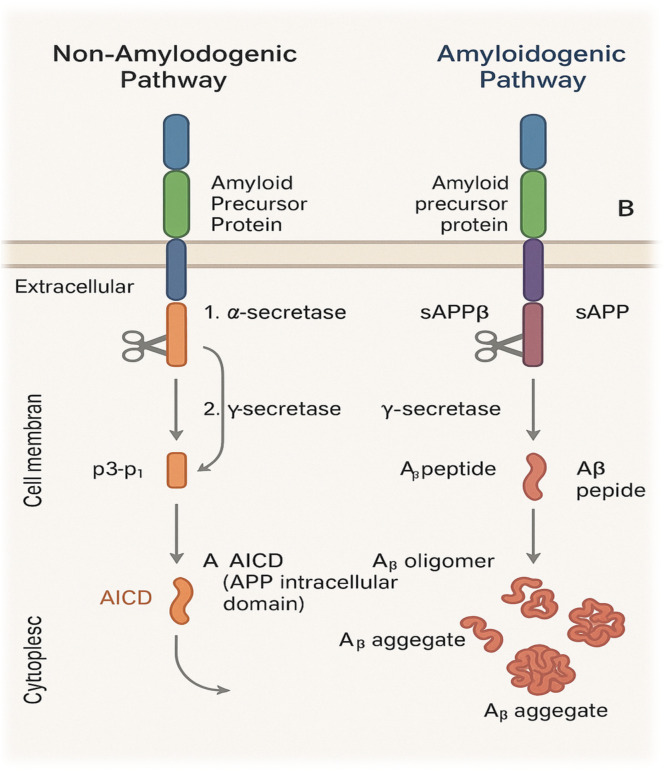
Amyloidogenic and non-Amyloidogenic pathways, Diagram illustrating both α- and β-secretase cleavage routes of the amyloid precursor protein (APP). The non-amyloidogenic pathway involves **α-secretase** cleavage yielding **sAPPα** and **CTF-α (C83)**, preventing Aβ formation. The amyloidogenic pathway involves **β-secretase (BACE1)** cleavage yielding **sAPPβ** and **CTF-β (C99)**, followed by **γ-secretase** cleavage to produce Aβ peptides (**Aβ₄₀** and **Aβ₄₂**), which aggregate into plaques.

**Fig. 3:**
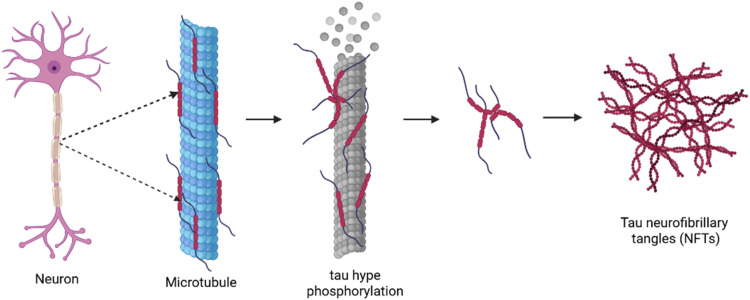
Neurofibrillary tangles (*NFT*_s_) pathways in *Alzheimers* disease, Representation of tau protein hyper phosphorylation leading to microtubule destabilization, synaptic loss, and neuronal death. Kinases such as **GSK-3β** and **CDK5** phosphorylate tau, causing it to detach from microtubules and form insoluble fibrillary aggregates.

**Fig. 4:**
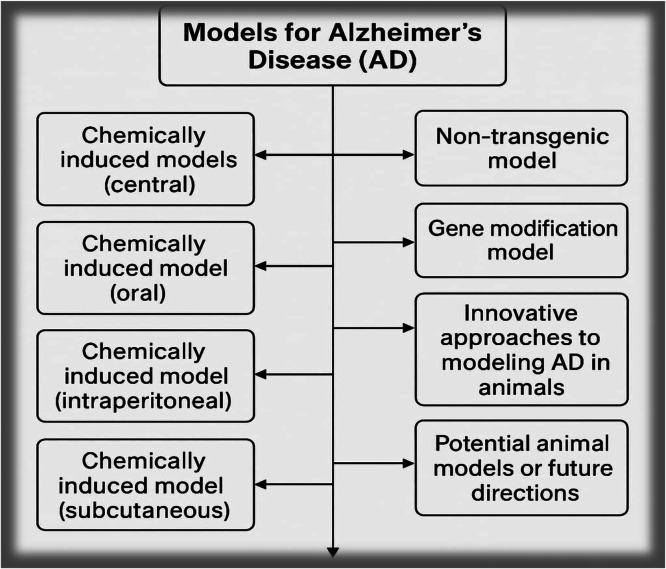
Preclinical Models for *Alzheimer’s disease,* chart summarizing commonly used experimental models of AD categorized by induction method: chemically induced (ICV-STZ, scopolamine, aluminum chloride, D-galactose), genetically engineered (3xTg, 5XFAD), and alternative models (zebrafish, C. elegans, Drosophila, non-human primates).

Conversely, in the **amyloidogenic pathway**, APP is first cleaved by **β-secretase (BACE1)** at the N-terminus of the Aβ region, producing **soluble APP-β (sAPPβ)** and **CTF-β (C99)**. Subsequent cleavage of CTF-β by **γ-secretase** generates **amyloid-β (Aβ)** peptides—predominantly Aβ₄₀ and Aβ₄₂—which tend to aggregate and form extracellular amyloid plaques characteristic of Alzheimer’s pathology [6]. The ratio of these isoforms is determined by secretase cleavage patterns. Under physiological conditions, their production and clearance are balanced. However, impaired Aβ degradation or clearance results in peptide accumulation, leading to oligomer formation, fibrillization, and extracellular plaque deposition. Mutations in APP, presenilin‑1 (PSEN1), or presenilin‑2 (PSEN2) genes further enhance amyloidogenic processing, increasing Aβ burden. Aβ oligomers exert potent neurotoxic effects, and amyloid plaques are frequently associated with activated glial cells and damaged neurons. Both soluble and aggregated Aβ species disrupt neuronal function, ultimately contributing to synaptic failure and neurodegeneration in AD [7]. (Figs. 2, 3, 4)

Neurofibrillary tangles primarily composed of hyperphosphorylated tau protein, which serves as a significant pathological marker in AD and plays a crucial role in its progression. Under normal conditions, tau protein helps maintain neuronal structure by stabilizing microtubules—tiny filaments essential for intracellular transport, communication, and overall neuronal function. In AD, however, tau undergoes abnormal chemical modification through a process called hyperphosphorylation.

This alteration causes tau to detach from microtubules and aggregate into insoluble tangles, leading to disruption of microtubule stability and transport, damage to neurons and their synaptic connections, Cognitive decline and worsening clinical symptoms.

Thus, tau pathology represents a central contributor to neurodegenerative changes and disease progression in Alzheimer’s disease [8].

Research reveals a relationship between tau and amyloid-beta in Alzheimer's pathology. The amyloid cascade hypothesis proposes that: Aβ buildup leads to abnormal tau protein changes (hyperphosphorylation) this, in turn, causes the tau protein to form clumps called neurofibrillary tangles, which damage brain cells. However, tau immunotherapy has shown potential in reducing amyloid and improving memory deficits in a transgenic model of mouse of AD, highlighting the significant role of tau pathologies in Aβ-induced neurotoxicity. The connection between tau and amyloid-beta (Aβ) pathologies in *Alzheimer’s disease* involves several factors, including: Calcium imbalance, certain enzymes (kinases) like GSK-3β and CDK-5, and changes in fatty acid levels. These factors can help development of *Alzheimer disease*. Furthermore, the combined effects of tau and Aβ-related pathologies lead to enhanced neurotoxicity and synaptic breakdown or dysfunction. While the evolution of tau-target therapies presents a promising avenue for Alzheimer's treatment, precise nature of the interaction between Amyloid betaβ and tau remains to be fully elucidated [5].

Additional neuropathological characteristics, including neuroinflammation, neuronal degeneration, oxidative stress, and synaptic dysfunction- and are believed to contribute to the progression of *Alzheimer’s disease*. These processes may interact, potentially exacerbating the underlying mechanisms and contributing to the development of AD. Animal models are crucial for exploring the etiology/pathogenesis of Alzheimer's disease and assessing potential treatments. Over the last several decades, numerous drug candidates have demonstrated encouraging anti-Alzheimer effects in preclinical studies, yet many have unexpectedly not succeeded in clinical trials. One possible explanation for this observation is suboptimal selection of preclinical models. Mammals like rodents have genetic physiological similarities to human beings. Although Animal models can provide valuable insights into *Alzheimer’s disease*, although they cannot fully replicate the human condition. Existing models can mimic some key features, and this review aims to summarize the characteristics of commonly used animal models in Alzheimer's research [[9], [10], [11], [12], [13], [14], [15]].

Researchers have conducted a range of studies using animal models to enhance the knowing of AD and to explore potential treatments or interventions. Animal models such as transgenic mice, are used to study *Alzheimer’s disease* by: introducing genes linked to AD (e.g., *APP* or presenilin), mimicking specific disease features, investigating disease mechanisms and testing potential treatments. Genetically modified mice with amyloid plaques are commonly used, some models involve injecting Aβ protein to induce plaques and inflammation, Models with abnormal tau protein are developed to study its role and investigate AD. Additionally, researchers examine how environmental factors, like certain toxins or chemicals (e.g., aluminum, pesticides), might contribute to AD development or progression in animal models. Chronic inflammation, alongside Aβ and *NFT*s, plays a important role in the pathogenesis of AD. Various agents have been utilized in AD models to trigger neuroinflammation and asses its effects on cognition and brain function [12].

This review outlines the different agents used in these models to replicate AD, including streptozotocin, scopolamine, aluminium chloride, trimethyltin and lipopolysaccharide, detailing their mechanism of action. It also highlights the findings from multiple studies employing these methods in the context of *Alzheimer’s* disease progression. Furthermore, based upon the Aβ hypothesis and the genetic factors associated with *Alzheimer’s*, there has been an increasing reliance on transgenic models that target amyloid plaques and tau proteins. It is important to note that the amyloid peptides produced in the mouse brain differ from those in the humans, and even despite amyloid deposition, mouse models often do not exhibit significant neuronal loss. While animal models for *Alzheimer’s disease* have limitations, but they can still provide valuable insights into the disease and potential treatments. This review aims to: Discuss the rationale behind choosing animal models for AD, examine current and emerging models, and assist researchers in choosing appropriate models for drug development and testing. The goal is to improve the effectiveness of preclinical models in understanding AD and developing treatments [[16], [17], [18], [19]].

## 2 Induction methods

### 2.1 Chemically induced models

#### 2.1.1 Intracerebroventricular-streptozotocin- induced Alzheimer’s model

Sporadic Alzheimer’s disease is influenced by genetics, environment, metabolism, and epigenetics. Early stages are often marked by disruptions in glucose metabolism and energy utilization, contributing to the disease's complexity. Animal models, such as the intracerebroventricular streptozotocin (ICV-STZ) model, 3 mg/kg in rats, help researchers understand Alzheimer’s disease by impairing insulin signaling in the brain, studying molecular mechanisms, and exploring potential treatments. This model contributes significantly to advancing Alzheimer’s research [[20], [21]].

The ICV-STZ model is used to study Alzheimer’s disease because it Disrupts brain insulin signaling, leading to oxidative stress, inflammation, & brain cell damage, causes memory and cognitive impairments, affects brain regions such as the hippocampus and cortex. Shows reduced expression of certain receptors, such as the α7-nicotinic acetylcholine receptor.

Researchers use this model to better understand Alzheimer’s pathology and to test potential treatments, including metformin, which may help mitigate disease progression [22].

#### 2.1.2 Amyloid-Induced Alzheimer’s model

As an alternative to the ICV-STZ model, amyloid-β peptides—including amyloid-β-42, amyloid-β-40, and amyloid-β-25–30—can be delivered via intracerebroventricular or intra-hippocampal injection. These amyloid variants differ in amino acid composition and possess varying levels of pathogenicity in Alzheimer’s disease, with Aβ-42 being the most pathogenic.

ICV administration of Aβ peptides induces neurodegeneration and impairs cognitive functions such as learning and memory. This is linked to oxidative and nitrosative stress. Injecting amyloid-beta (Aβ) into the brain causes brain cell damage, memory deficits, learning impairments, and increased oxidative stress, thereby helping researchers understand Alzheimer’s disease mechanisms.

Amyloid-β increases the production of harmful molecules called reactive oxygen species (ROS). In an Aβ-42 model of Alzheimer’s, amyloid precursor protein (APP) processing is enhanced, leading to greater accumulation of senile plaques. Amyloid also affects brain receptors, such as nicotinic acetylcholine receptors, disrupts normal brain function (cholinergic dysfunction), and plays a significant role in AD models [24].

Central administration of amyloid-beta is associated with the development of tau tangles, and mitochondrial dysfunction—considered the powerhouse of the cell—may occur through oxidative pathways. As primary pathological features of AD, amyloid peptides are targeted by monoclonal antibodies such as aducanumab, which has recently received approval from the US FDA for AD therapy. This approval is noteworthy compared to traditional AD treatments, as aducanumab functions as an anti-amyloid-β agent, potentially halting disease progression and neurodegeneration [[21], [22], [23], [24], [25], [26], [27], [28], [29]].

### 2.2 Intracerebroventricular administration models

#### 2.2.1 Alzheimer's model using colchicine

Colchicine is a medication derived from the *Colchicum autumnale* plant and is commonly used to treat gout. Its mechanism of action involves binding to tubulin and affecting microtubule dynamics. Although generally administered orally for therapeutic purposes, in experimental models, a low dose of colchicine (15 μg) injected intracerebroventricularly in rats induces cognitive impairments similar to those observed in Alzheimer’s disease.

This model leads to disruption of oxidative balance, damage to cholinergic pathways, Increased neuroinflammation, Resultant brain cell damage and dysfunction.

Colchicine promotes inflammation and neuronal damage by increasing inflammatory mediators such as COX-2, PGE2, IL-1β, and TNF-α. It also disrupts microtubule integrity, ultimately resulting in neuronal death, which contributes to neurodegeneration [30].

### 2.3 Intraperitoneal administration models

#### 2.3.1 Alzheimer's model using scopolamine

Scopolamine, also known as hyoscine, is a tropane alkaloid and a potent anticholinergic agent derived from *Hyoscyamus niger*. It is commonly used to prevent motion sickness and manage post-surgical nausea. As a competitive antagonist of muscarinic receptors, scopolamine effectively addresses various cholinergic-related symptoms such as increased bowel movements, salivation, lacrimation, and sweating.

Acetylcholine plays a vital role in memory formation by enhancing synaptic connections. Therefore, the cholinergic blockade induced by scopolamine is widely used as an experimental model for Alzheimer’s disease. Scopolamine increases acetylcholinesterase (AChE) activity, accelerating the breakdown of acetylcholine [[31], [32], [33], [34]].

In research, a dose of 2 mg/kg administered intraperitoneally is commonly used to mimic Alzheimer’s disease. This disrupts brain connectivity, impairing spatial memory and affecting brain networks. The scopolamine model offers advantages such as not requiring complex brain surgery and being effective for testing treatments that boost cholinergic function (e.g., donepezil, rivastigmine) and reduce oxidative stress (e.g., melatonin). This makes it a valuable tool for studying Alzheimer's prevention and treatment strategies.

#### 2.3.2 Alzheimer's model using atropine

Atropine is a drug derived from the alkaloid source Atropa belladonna and is utilized as an anticholinergic agent for the treatment of bradycardia and myopia. Similar to scopolamine, atropine interferes with the cholinergic system by diminishing the activity of muscarinic acetylcholine receptors. It also exerts a minor inhibitory effect on nicotinic receptors.

Administration of atropine at a dose of 5 mg/kg intraperitoneally for 21 days has been shown to induce the formation of amyloid plaques, which are characteristic features of Alzheimer’s disease. This process may be linked to the relationship between cholinergic signaling and amyloid production. Additionally, a reciprocal reduction in acetylcholine release due to amyloid-beta has been observed [35].

#### 2.3.3 Alzheimer’s model using aluminum chloride (AlCl_3_)

Aluminum toxicity is used to model Alzheimer’s disease in animals through specific dosing and duration. Excessive aluminum exposure results in toxicity. Intraperitoneal injections of AlCl₃ at 4 mg/kg for 40 days induce an Alzheimer’s-like model in rodents. The key pathological mechanisms in the AlCl₃ model involve oxidative stress and mitochondrial damage, primarily through inhibition of NADH dehydrogenase. These processes contribute to Alzheimer’s-like pathology.

The AlCl₃ model exhibits damage to memory-related brain areas such as the cortex and hippocampus, increased neuroinflammation indicated by markers like iNOS, NF-κB, COX-2, and various proinflammatory cytokines, leading to neurodegeneration and neurotoxicity. Aluminum salts induce cholinergic dysfunction and oxidative stress, which result in apoptosis and neuronal cell death.

This model is particularly relevant for studying Alzheimer’s disease-like pathology and is favored for evaluating prophylactic interventions aimed at prevention rather than only symptomatic protection. It facilitates the development of therapeutic agents targeting disease prevention [[31], [32], [33], [34], [35]].

### 2.4 Subcutaneous administration models

#### 2.4.1 Galactose-induced aging model

D-Galactose, a sugar found in the brain, is present in various dietary sources such as dairy products, avocados, and sugar beets. It is metabolized into reactive oxygen species (ROS). For instance, milk contains approximately 7.12 mg/100 g of galactose, avocados contain 0.66 g sugar/100 g (including galactose), and sugar beets contain about 0.65 % galactose.

Studies have demonstrated that subcutaneous administration of D-galactose at doses of 50, 100, and 200 mg/kg leads to increased latency in locating platforms in the Morris water maze (MWM) test and reduced recognition of new objects in the novel object recognition (NOR) test, indicating a dose-dependent decline in memory. This treatment also increases oxidative damage and stress levels in the hippocampus.

Moreover, D-galactose administration has been shown to weaken the immune system, simulating age-related brain decline. It reduces neurogenesis in the dentate gyrus and disrupts calcium homeostasis in the brain cortex, resulting in neuronal overexcitation and cell death akin to dementia pathology. This chemical model is particularly relevant for studying Alzheimer’s disease cases linked to insulin resistance, as D-galactose induces insulin resistance [36], [37], [38], [39].

### 2.5 Gene-modified model/ genetically engineered model

#### 2.5.1 Three-gene transgenic model

This model combines three genetic mutations—APP, presenilin 1 (PS1), and mutant tau protein (p-tau)—to replicate Alzheimer’s pathology in mice. Referred to as the triple transgenic model, these gene mutations impact Alzheimer’s development, as APP and tau proteins are associated with amyloid plaques and neurofibrillary tangles, while PS1 is part of the γ-secretase complex that processes APP.

To create this model, scientists microinject transgenes for the APP-Swe, PS1-M146V, and tau-P301L mutations into mice, generating a familial Alzheimer’s disease model. This transgenic model exhibits brain atrophy, synaptic damage, and neuronal loss, with no capacity for neuronal regeneration in key brain regions, leading to cognitive and memory deficits. Cognitive functions involving spatial navigation and object recognition are impaired in these mice.

Besides cognitive problems, other physical changes have been observed in the mutated mice. Modified mice with mutations in the amyloid precursor protein and PS2 on chromosome 1, or only in APP, have also been created, showing traits similar to familial Alzheimer’s disease. While APP-based models facilitate research into certain treatments, they lack the ability to fully model the most prevalent sporadic form of Alzheimer’s disease [[40], [41]].

#### 2.5.2 Five-mutation Alzheimer's model (5XFAD)

The 5XFAD model is a transgenic mouse model of familial Alzheimer’s disease that carries five mutations driving amyloid plaque formation, gliosis, synaptic damage, and neuronal loss. The model expresses the APP695 protein with the Swedish (K670N/M671L), Florida (I716V), and London (V717I) mutations. Additionally, it harbors two mutations in the presenilin-1 (PS1) gene: M146L and L286V.

The 5XFAD model develops Alzheimer’s-like pathology early, exhibiting prominent amyloid plaques, neuroinflammation, and neurodegeneration, particularly in the cortex and hippocampus. This model is widely used to study Alzheimer’s disease mechanisms and to evaluate potential treatments [[41], [42], [43], [44], [45]].

### 2.6 Naturally occurring disease model or non-transgenic model

#### 2.6.1 Model of aged rodent/rats

As rats age, they experience a natural decline in brain regions such as the hippocampus, leading to learning and memory deficits. This model is preferred over chemically induced models because it is non-invasive and better reflects the symptoms of late-onset or sporadic Alzheimer’s disease. Rats aged between 15 and 20 months are typically used for this model, making it more clinically relevant to the disease.

Age-related dementia involves neuroinflammation, oxidative stress, and metabolic disruptions, which are further exacerbated by factors such as obesity and physical inactivity. Alzheimer’s-like pathology is evident in these aged rodents. However, interventions such as exercise and intermittent fasting show promise in reversing damage, improving brain function, and enhancing memory [46].

#### 2.6.2 Diet-induced model

Researchers use high-fat diets to model and study obesity, insulin resistance, and type 2 diabetes. Recent studies suggest that high-fat diets may also serve as models for cognitive dysfunction. Feeding rats or mice a fat-rich diet for approximately 10 to 14 weeks can lead to central insulin resistance, in addition to peripheral insulin sensitivity impairment. This diet typically consists of approximately 25 % fat, 20 % protein, and 50 % carbohydrates.

Alzheimer’s disease negatively impacts brain insulin function. This model highlights insulin resistance, which is critical for assessing memory deficits and developing treatments.

Additionally, obesity resulting from a high-fat diet can reduce cerebral blood flow, lowering oxygen and glucose supply to the brain, which may contribute to vascular dementia. Cognitive decline associated with hypertension and diabetes is also linked to high dietary fat intake. Cholesterol derived from fat may promote the formation of senile plaques by increasing amyloid precursor protein (APP), leading to neuronal loss. Other factors contributing to memory loss include disrupted lipid balance and impaired glucose transport.

The high-fat diet model exacerbates neuroinflammation and oxidative stress by decreasing antioxidant defenses and increasing pro-inflammatory signals [[47], [48], [49]].

### 2.7 Alternative animal models beyond mice and rats

#### 2.7.1 Zebrafish research model

Zebrafish, a tropical freshwater fish, are a valuable research model due to their transparent embryos, which allow easy observation of development, and their genetic similarity to humans, enabling the study of disease mechanisms. Identification of mutant genes linked to Alzheimer’s disease, such as APP and presenilin, has made zebrafish a useful model for studying familial Alzheimer’s disease (FAD).

Their unique characteristics make zebrafish an attractive model organism for developmental biology, genetics, and disease modeling. Compared to rodent models, zebrafish have fewer neurons, which aids in understanding neuronal networks, exhibit rapid neurogenesis, and have significant reproductive capacities, along with ease of genetic modification. Their small size, simple tissue layout, and high fertility make them ideal for drug testing.

However, there are some downsides, such as higher mortality rates, challenges in maintenance, and less physiological similarity to humans compared to rodents. In addition to natural mutations, scientists have also created artificial mutations in zebrafish using techniques such as zinc finger nucleases, TALENs, and CRISPR. These methods allow targeted genetic modifications to aid research on neurodegenerative diseases [[48], [49], [50]].

#### 2.7.2 Nematode worm model (C. elegans)

*Caenorhabditis elegans (C. elegans*) is a nematode worm widely used in recent years to study human diseases, particularly neurodegenerative disorders. Its anatomy is simpler and more transparent than that of rodents and humans. About 40 % of its genes are homologous to human genes related to amyloid protein and tau protein, which are critical in Alzheimer’s research, making it suitable for genomic studies related to AD.

This model offers several advantages, including high reproduction rates, low dietary requirements, and neurons that can be easily observed under a microscope. Despite these benefits, it also has some drawbacks, such as a short lifespan and small size, which can make handling challenging.

*C. elegans* is an effective model for assessing cognitive functions, such as spatial memory. Furthermore, it allows manipulation of synapses to study memory and synaptic function. Transgenic C. elegans models have been developed to explore amyloid formation in the context of familial Alzheimer’s disease [51].

#### 2.7.3 Fruit fly model

*Drosophila melanogaster* is a valuable model for studying Alzheimer's disease (AD), as it can mimic late-onset AD symptoms through the expression of proteins such as APP, BACE-1, and tau. These proteins lead to amyloid accumulation and neurodegeneration. The tau ortholog in Drosophila refers to genes analogous to human tau proteins. Presenilin (PS), a component of the γ-secretase complex, helps process proteins like APP, and mutations in this complex can contribute to AD-related pathology.

This model is also useful for studying other neurodegenerative diseases due to Drosophila’s short lifespan and rapid reproduction. The Drosophila genome contains genes such as APPl and dBACE, along with the enzyme γ-secretase, providing a valuable system for studying amyloid-related mechanisms and memory processes relevant to Alzheimer’s disease. Additionally, genetically modified Drosophila models that overproduce Aβ in the central nervous system have been instrumental in studying familial Alzheimer’s disease [[52], [53], [54]].

#### 2.7.4 Cavia porcellus model

Unlike common rodent models, guinea pigs (*Cavia porcellus*) possess an amyloid-beta (Aβ) peptide sequence identical to humans, making them a valuable non-transgenic model for Alzheimer’s disease research. High-cholesterol diets have been shown to influence enzyme activity in guinea pigs, leading to increased Aβ production.

The guinea pig model offers distinct advantages for studying Alzheimer’s disease due to its tau gene structure and cholesterol-influenced isoform ratio, which closely mirror human conditions. Notably, guinea pigs are the only small animal model in which the generation of the PS2V isoform has been observed. PS2V is a truncated isoform of presenilin 2 that has been linked to increased γ-secretase activity and Aβ synthesis, and it is found at elevated levels in the brains of individuals with sporadic Alzheimer’s disease [[56], [57], [58], [59]].

The similarities between guinea pigs and humans at the genetic and biochemical levels highlight the suitability of this model for analyzing the impact of dietary factors such as cholesterol on the regulation of Alzheimer’s disease-related genes.

#### 2.7.5 Non-human primate (NHP) models (monkey)

Non-human primate (NHP) models, using monkeys, are valuable for Alzheimer’s disease research due to their close brain similarities to humans. These models aid in understanding disease progression, amyloid plaque formation, and cognitive decline. NHP models are also instrumental in testing potential treatments, providing valuable insights for human Alzheimer’s research.

To date, amyloid deposition in brain tissue has been documented in a wide range of non-human primates, including rhesus monkeys, chimpanzees, vervet monkeys, marmosets, and cynomolgus monkeys [[55], [56], [57], [58], [59], [60]].

### 2.8 *In-vitro* models

*In vitro* models provide controlled cellular and molecular insights into disease mechanisms but are less comprehensive than *in vivo* models, which capture the full biological context.

#### 2.8.1 Neuroblastoma model

The SH-SY5Y cell line, derived from neuroblastoma through sub cloning techniques, has the potential to differentiate into neuronal cells exhibiting functions similar to mature neurons when exposed to appropriate treatments. Neuroblastoma comprises both Schwann cells and neuroblasts, making SH-SY5Y cells valuable for developing potential therapeutic agents aimed at treating Alzheimer’s disease [61].

#### 2.8.2 Induced pluripotent stem cell (iPSC)-derived cells

By reprogramming somatic cells from Alzheimer’s patients into induced pluripotent stem cells (iPSCs) and differentiating them into neurons, scientists can create models that closely resemble the human disease, enabling in-depth study of Alzheimer’s disease (AD) mechanisms. Alzheimer’s iPSC models can replicate key pathological features such as amyloid plaques and neurofibrillary tangles, allowing researchers to investigate disease mechanisms and test potential treatments for both familial and sporadic forms of AD [[62], [63], [64], [65], [66]].

### 2.9 Innovative approaches to modeling Alzheimer’s disease in animals

#### 2.9.1 Streptozotocin (STZ)-induced Alzheimer's model in APP/PS1 mice

The STZ-APP/PS1 model combines chemically induced dementia via Streptozotocin (STZ) with genetic modifications (APP/PS1) to provide a novel approach for studying memory loss and Alzheimer’s disease in mice. It is reasonable to anticipate that this model will exhibit a greater number and similarity of Alzheimer’s disease (AD) characteristics, potentially offering synergistic effects compared to the standalone STZ or APP/PS1 models. Additionally, this newly developed animal model is likely to exhibit overlapping features of both sporadic and familial forms of AD. In this model, STZ is injected directly into the brain ventricles (intracerebroventricular, ICV) of APP/PS1 mutant mice [67].

#### 2.9.2 Fructose-fed model

Fructose consumption can cause insulin resistance and disrupt glucose metabolism. Additionally, fructose can trigger the release of glucocorticoid hormones, which play a role in regulating food intake. Excessive dietary intake of fructose may result in neuronal damage, particularly affecting brain regions associated with hunger and food intake, such as the hypothalamus and hippocampus. This can ultimately lead to memory deficits and cognitive dysfunction.

Recent studies suggest that Alzheimer’s disease is increasingly being recognized as a metabolic condition, characterized by disrupted glucose metabolism and impaired insulin signaling in the brain [68].

#### 2.9.3 Hypertension-based model for studying Alzheimer's

Elevated blood pressure (hypertension) refers to the excessive force applied to the walls of arteries and veins. This condition can impact various organs, including the brain, as blood is responsible for delivering oxygen and essential nutrients necessary for cell survival. Chronic hypertension can impede blood flow to the brain, limiting its access to vital nutrients and oxygen.

High blood pressure in the body can be accompanied by decreased cerebral blood flow. In animal studies, hypertension can be induced through pharmacological means or lifestyle modifications such as reduced physical activity, high-sodium diets, or consumption of cholesterol-rich foods. Research suggests that hypertension may lead to cognitive decline in rats, potentially contributing to Alzheimer’s-like symptoms, and it can be used to enhance existing Alzheimer’s disease models [[67], [68], [69]].

#### 2.9.4 Recent advances

iPSC-derived neurons and brain organoids provide human-specific systems to replicate AD pathology. Integration with single-cell multi-omics and high-resolution neuroimaging allows for spatiotemporal mapping of neuronal degeneration, while optogenetics offers real-time modulation of synaptic activity in animal models, bridging preclinical and clinical research.

##### 2.9.4.1 Brain organoids and induced pluripotent stem cells (iPSCs)

From the somatic cells of the patient neurons and glial cells were collected, which shows numerous characteristics such as impaired synaptic function, anomalous calcium signaling, accumulation of amyloid-β, and tau hyperphosphorylation. These models aid scientists to understand the disease impact on genetic factors such as APOE4 and alterations in APP, PSEN1, and PSEN2. Three-dimensional brain organoids enhance these capabilities by generating complicate microenvironments that replicate the architecture of the cortex, the dynamics of synaptic networks, and the interactions between glial and neuronal cells. Organoids can display early signs of Alzheimer's disease, such as the development of amyloid, tau, and networks that aren't stable [70,71].

##### 2.9.4.2 Single cell multi-omics

Techniques such as scRNA-seq, ATAC-seq, proteomics, and metabolomics are employed to identify cellular states, inflammation, fragile neuronal clusters, disease-associated microglia (DAM), and excitatory neurons in the entorhinal cortex, modifications in transcriptional, epigenetic, and metabolic pathways throughout disease progression can be integrated with multi-omic analysis. Researchers can utilize these techniques on human tissue, brain organoids, and models derived from induced pluripotent stem cells to construct molecular timelines that illustrate the progressive decline associated with Alzheimer's disease. Single-cell multi-omics can also help to find new drugs, biomarkers, and treatment targets for AD. These tools help identify distinct cellular traits in each patient, which improves precision medicine [72,73].

##### 2.9.4.3 High-resolution neuroimaging techniques

Neuroimaging is highly important for early diagnosis, to know disease progression and finding out how healthy new treatments perform. The sophisticated modalities enable the imaging of AD pathology includes 7TMRI, diffusion tensor imaging, functional MRI, amyloid and tau PET, these can show a significant relationship between neurodegeneration and cognitive impairment. Deterioration memory and executive function circuits can be imaged and also reveals the reduction in size of the hippocampus and the thinning of the cortex. by magnetic resonance imaging (fMRI). iPSC and single-cell data approaches facilitate the correlation between molecular alterations and overall brain function modifications. Connectome mapping, super-resolution microscopy, and diffusion kurtosis imaging advanced imaging techniques are used to reveal changes at the network and microstructural levels [74].

##### 2.9.4.4 Contemporary biological models - microfluidic "brain-on-a-chip"

This includes how neurons communicate signals, how synapses connect, and how toxic substances like amyloid and tau spread. By constructing isogenic lines with CRISPR/Cas9, scientists can study the function of specific genes and observe how cells of living organisms function through the use of chimera models, which allow scientists to transplant human neurons or microglia into mouse brains. These revolutionary tools are filling in the gaps left by older models and connecting basic cell models to complex human disorders [75,77].

##### 2.9.4.5 Computer approaches

AI and ML Better machine learning and artificial intelligence are changing how we learn about and treat Alzheimer's disease. In fundamental research, artificial intelligence (AI) sorts patients by molecular subtype based on data from imaging, electrophysiological, and omics. It also finds out which systems cause diseases. We can learn more about how diseases spread, how well therapies work, and how to find new drugs that might work by utilizing predictive models. Machine learning helps doctors make better diagnoses by recognizing little changes in speech, retinal imaging, physical movement, or MRI that could happen years before a person's mental health gets worse. Wearables and AI-powered cell phones may now gather digital data that lets you check your health and risk in real time. These computer-based tools help us find things faster, tailor treatments to each person, and get around problems with previous diagnostic methods. Tele-neurology platforms, remote monitoring tools, and digital health tools are some of the new technologies that are speeding up care for Alzheimer's patients. Wearable sensors and smartphone apps can help you keep track of your sleep, thoughts, and physical activity over time. This makes it easy to find problems early and adjust how you help. AI-assisted cognitive testing makes it possible to do a large-scale, fair assessment of cognitive ability. There are many benefits of using telemedicine, such as better access to specialists, more information for caregivers, and more consistent care. You can do cognitive training and behavioral interventions at home as part of a digital therapy program. They are inexpensive methods to feel better. These new ways of doing things are based on care models that are proactive, focus on the individual, and are easy to reach. Tools that help without hurting There is increasing optimism over the advancement of non-invasive neuromodulation technology for patient treatment. Animal studies have demonstrated that gamma-frequency (40 Hz) sound or light therapy can diminish amyloid and tau disease by synchronizing brain waves. Preliminary human studies have shown enhancements in cognitive function and network connectivity [74,76,77].

##### 2.9.4.6 Transcranial direct current stimulation (tDCS) and transcranial magnetic stimulation (TMS)

TMS enhances synaptic plasticity and cognitive performance, while tDCS modulates cortical excitability. Focused ultrasound temporarily disrupts the blood-brain barrier, allowing medicines to pass. These devices may work with existing Alzheimer's medications and offer a fresh approach. Nineth Area: Alzheimer's and Optogenetics Research Optogenetics helps scientists control glial and neuron behavior. They can determine which circuits Alzheimer's affects this way. Researchers used light to wake up "engram" cells in sick mice to restore memory. Optogenetics has revealed how Alzheimer's disease alters microglia function, gamma oscillations, and the hippocampus-entorhinal cortex relationship. Even though optogenetics research is young, it is already developing novel brain-affecting medications and gadgets. This technology can help us understand how elevated tau and amyloid levels alter brain circuits. ML and AI are changing Alzheimer's disease research and treatment. AI classifies patients by genetic subtypes utilizing imaging, electrophysiological, and omics data in basic research. It determines which systems cause illness. We can learn how diseases spread, how well treatments work, and how to find new drugs using predictive models. Machine learning helps clinicians diagnose mental health issues by detecting small changes in speech, retinal imaging, physical movement, or MRI years before they worsen. Real-time health and risk assessment is possible with AI-enabled wearables and phones. These computer technologies help us find things faster, adapt therapies, and avoid problems with earlier diagnostic methods. Tele-neurology platforms, remote monitoring tools, and digital health tools are speeding Alzheimer's care. Track your sleep, thoughts, and physical activities using wearable sensors and smartphone apps. You can spot issues early and adjust your aid. AI allows for fair and large-scale cognitive testing. Telemedicine makes it easier for caregivers to acquire information, experts to see patients, and care more consistent. Home cognitive training and behavioral treatments are part of digital therapy. Cheap methods to feel better. These new strategies use proactive, person-centered, and accessible care paradigms. Helpful tools without harm Patient treatment with non-invasive neuromodulation technologies is gaining optimism. Brain wave synchronization with gamma-frequency (40 Hz) sound or light therapy reduces amyloid and tau disease in animals. Cognitive function and network linkage improved in early human studies [78,79].

### 2.10 Potential animal models on the horizon/future directions

While the previously discussed animal-based research models partially reflect Alzheimer’s disease (AD) pathology, they do not fully replicate the clinical manifestations associated with the condition. Consequently, exploring alternative approaches may lead to a deeper understanding of AD-related pathological processes. Several strategies are worth considering, although these models have not been thoroughly examined yet. They present potential avenues for future research aimed at developing more effective AD models and addressing the limitations noted earlier. Additionally, these models may help identify new targets for Alzheimer's treatments [[79], [80]].

#### 2.10.1 Model of Alzheimer’s disease induced by alloxan

A dose of 160 mg/kg of alloxan destroys pancreatic beta cells, impairing insulin secretion. Previous research has primarily focused on alloxan in relation to diabetes models. Given that Alzheimer’s disease (AD) exhibits characteristics similar to diabetes, such as insulin resistance, alloxan has potential for use in studying its effects on inducing insulin resistance in the brain when delivered directly to this organ rather than through peripheral administration [79].

Alloxan’s ability to produce reactive oxygen species (ROS) may drive neurodegeneration. Additionally, its lower cost and wider availability compared to streptozotocin (STZ) make it a promising candidate for future Alzheimer’s disease modeling studies [81].

#### 2.10.2 Acetylcholinesterase-linked Alzheimer’s disease model

Acetylcholinesterase (AChE) is an enzyme that degrades acetylcholine, a neurotransmitter vital for cognitive functions and memory throughout the brain. Acetylcholinesterase, along with its analogs and activators such as pralidoxime and obidoxime, can be delivered to brain regions associated with memory management and cognitive operations. Pralidoxime and obidoxime are primarily utilized in the treatment of organophosphate poisoning and nerve agent exposure.

The role of AChE in Alzheimer's disease is significant, as it influences acetylcholine levels, which are crucial for cholinergic function. Therapeutic strategies often target AChE to inhibit its activity, thereby enhancing cholinergic transmission and improving cognitive symptoms in AD patients [[82], [83], [84], [85], [86], [87]].

#### 2.10.3 Neurodegeneration model via lesion induction

Brain lesions, particularly in regions such as the cerebral cortex and hippocampus, can cause cognitive impairments and memory loss that mimic dementia symptoms. Bilateral lesions in the hippocampus are associated with deficits in learning. Additionally, significant damage has been observed with radiofrequency lesioning. The nerve cell injury resulting from these lesions may ultimately contribute to neurodegeneration.

Neurodegeneration in brain areas responsible for cognition can serve as a secondary contributor to the pathology of Alzheimer’s disease. Furthermore, targeted damage to specific brain regions can disrupt particular types of memory, affecting both spatial and recognition memory. However, these highly invasive techniques raise serious ethical concerns and carry risks of irreversible brain damage, with potentially fatal outcomes in extreme cases [[88], [89], [90], [91], [92]].

### 2.11 Emerging models: optogenetic and viral vector-based systems

Optogenetic activation of amyloidogenic pathways and viral vector-mediated delivery of mutated human genes (e.g., APP, MAPT) are cutting-edge methods that replicate AD-like pathology in animal brains. These models enable spatially and temporally controlled induction of plaques and tangles, providing high translational relevance and insight into disease mechanisms [93].

## 3 Conclusion

Alzheimer’s disease remains one of the most formidable challenges in neuroscience, with its multifactorial etiology, intricate pathology, and devastating societal impact. Over the decades, animal models have served as indispensable tools, offering vital windows into the mechanisms underlying amyloid pathology, tau aggregation, neuroinflammation, oxidative stress, and synaptic dysfunction. Yet, as highlighted throughout this review, no single model fully recapitulates the complexity of human Alzheimer’s disease. Each carries its own strengths and limitations, reflecting only fragments of the broader pathological landscape.

The progression from traditional chemically induced and transgenic rodent models to innovative platforms including zebrafish, fruit flies, nematodes, guinea pigs, and non-human primates has greatly enriched our ability to study distinct facets of the disease. Furthermore, the emergence of stem cell derived neural organoids, hybrid chemical-genetic models, and integrative metabolic paradigms represents a paradigm shift, bridging gaps between preclinical experimentation and human pathology. These approaches not only allow for a more faithful reconstruction of disease progression but also provide invaluable platforms for precision drug testing, biomarker discovery and personalized therapeutic intervention.

Importantly, the future of Alzheimer’s research lies in embracing a multimodal and integrative strategy: combining genetically engineered models with metabolic and environmental stressors, harnessing the power of iPSC-derived neurons and organoids, and applying advanced technologies such as single-cell multi-omics, high-resolution neuroimaging, and optogenetics. These technologies show how molecules interact and how brain neural networks work. More specific therapies, faster disease detection, and better classification are feasible. Together, these technologies will accelerate precision medicine. Ethical considerations must also remain central, particularly when employing sentient animal models or manipulating cognition in higher species.

In conclusion, while no perfect model exists, the convergence of diverse experimental systems holds the promise of unraveling the enigma of Alzheimer’s disease. By refining these models and aligning them more closely with human pathology, we can accelerate the translation of bench side discoveries into bedside therapies. Ultimately, the quest for more representative models is not only a scientific necessity but also a moral imperative—paving the way toward effective interventions that may one day alleviate the immense burden of Alzheimer’s disease on individual’s families and society at large.

## Funding

Not Applicable

## Declaration of the use of generative AI and AI-Assisted technologies

The authors declare that generative artificial intelligence (AI) and/or AI-assisted tools were used only to improve the language, grammar, and readability of the manuscript**.** These tools were not used to generate scientific content, analyze data, or alter the interpretation of published findings. The authors retain full responsibility for the accuracy, originality, and integrity of the content.

## Ethical statement

This article is a review of previously published studies. Therefore, no ethical approval was required.

## Studies on human and/or animal subjects

This review article did not involve any studies with human participants or animals performed by the authors.

## Informed consent

Not applicable, as this study did not involve human participants.

## Ethical approval / registration number

Not applicable.

## CRediT authorship contribution statement

**Nadeemullah Khan:** Writing – original draft, Writing – review & editing. **Somnath De:** Writing – review & editing, Supervision, Conceptualization. **Suhasini Boddu:** Writing – review & editing. **Navya Pravala:** Writing – review & editing.

## Declaration of competing interest

The authors declare the following financial interests/personal relationships which may be considered as potential competing interests:

Somnath De reports writing assistance was provided by St.Pauls College of Pharmacy. Somnath De reports a relationship with St.Pauls College of Pharmacy that includes: board membership. There is No conflict of interest If there are other authors, they declare that they have no known competing financial interests or personal relationships that could have appeared to influence the work reported in this paper.
